# Comparison of phasor analysis and biexponential decay curve fitting of autofluorescence lifetime imaging data for machine learning prediction of cellular phenotypes

**DOI:** 10.3389/fbinf.2023.1210157

**Published:** 2023-06-29

**Authors:** Linghao Hu, Blanche Ter Hofstede, Dhavan Sharma, Feng Zhao, Alex J. Walsh

**Affiliations:** Department of Biomedical Engineering, Texas A&M University, College Station, TX, United States

**Keywords:** autofluorescence, fluorescence lifetime, machine learning, metabolism, macrophage

## Abstract

**Introduction:** Autofluorescence imaging of the coenzymes reduced nicotinamide (phosphate) dinucleotide (NAD(P)H) and oxidized flavin adenine dinucleotide (FAD) provides a label-free method to detect cellular metabolism and phenotypes. Time-domain fluorescence lifetime data can be analyzed by exponential decay fitting to extract fluorescence lifetimes or by a fit-free phasor transformation to compute phasor coordinates.

**Methods:** Here, fluorescence lifetime data analysis by biexponential decay curve fitting is compared with phasor coordinate analysis as input data to machine learning models to predict cell phenotypes. Glycolysis and oxidative phosphorylation of MCF7 breast cancer cells were chemically inhibited with 2-deoxy-d-glucose and sodium cyanide, respectively; and fluorescence lifetime images of NAD(P)H and FAD were obtained using a multiphoton microscope.

**Results:** Machine learning algorithms built from either the extracted lifetime values or phasor coordinates predict MCF7 metabolism with a high accuracy (∼88%). Similarly, fluorescence lifetime images of M0, M1, and M2 macrophages were acquired and analyzed by decay fitting and phasor analysis. Machine learning models trained with features from curve fitting discriminate different macrophage phenotypes with improved performance over models trained using only phasor coordinates.

**Discussion:** Altogether, the results demonstrate that both curve fitting and phasor analysis of autofluorescence lifetime images can be used in machine learning models for classification of cell phenotype from the lifetime data.

## 1 Introduction

The fluorescence lifetime is a quantitative measurement of the time a fluorophore takes to relax to the ground state after being excited by a photon. Fluorescence lifetime imaging (FLIM) is a nondestructive imaging technique that has high sensitivity to fluorophore microenvironment and molecular conformation. The endogenous fluorophores reduced nicotinamide adenine dinucleotide (NADH) and flavin adenine dinucleotide (FAD) are two critical coenzymes involved in different cellular metabolic reactions such as oxidative phosphorylation (OXPHOS) and glycolysis. Autofluorescence imaging of these coenzymes provides information about the metabolic state of cells without using labels or cell fixation (Chance et al., 1979; Georgakoudi and Quinn, 2012). NADH and its phosphorylated form NADPH, exhibit the same spectral properties; therefore, NAD(P)H represents the combined fluorescence of both molecules (Huang et al., 2002). NAD(P)H and FAD molecules both have two conformations: either free or protein-bound, each of which has a different lifetime. Free NAD(P)H has a short lifetime, and protein-bound NAD(P)H has a long lifetime (Lakowicz et al., 1992). FAD has a long lifetime when free and a short lifetime when bound to a protein (Nakashima et al., 1980).

FLIM of NAD(P)H and FAD and subsequent quantitative analysis is sensitive to metabolic changes within cells due to alterations in cellular metabolic activity or cellular functional phenotypes. For example, macrophages, which play a critical role in the innate immune response, can polarize into either a pro-inflammatory, “classically-activated” phenotype (M1) or an anti-inflammatory and pro-wound healing, “alternatively-activated” (M2) phenotype (Mosser and Edwards, 2008; Galvan-Pena and O'Neill, 2014). M1 and M2 macrophages are dependent on different metabolic activities within the cell (Galvan-Pena and O'Neill, 2014). These metabolic differences between M1 and M2 macrophages can be detected using FLIM of NAD(P)H and FAD, allowing for the differentiation of macrophage subtypes without using fluorescent tags (Alfonso-Garcia et al., 2016; Borowczyk et al., 2020; Heaster et al., 2020; Heaster et al., 2021; Miskolci et al., 2022). Similarly, cancer cells are often characterized by aerobic glycolysis (Warburg, 1956), and exhibit metabolic perturbations when exposed to chemotherapy. FLIM of NAD(P)H and FAD has been used to investigate metabolic differences between non-cancer and cancerous cells and to track metabolic changes due to anti-cancer drug response (Bird et al., 2005; Walsh et al., 2013; Sharick et al., 2018). Machine learning prediction models that use the quantitative information obtained from FLIM are a promising technique to identify functional cell phenotypes such as drug-responsive or resistant cancer cells (Cardona and Walsh, 2022), activated or quiescent T cells (Walsh et al., 2021), M1 or M2 macrophages (Neto et al., 2022), and differentiation efficiency of cardiomyocytes from stem cells (Qian et al., 2021).

FLIM data can be analyzed by several methods including exponential decay fitting and phasor analysis. In time domain FLIM, a short laser pulse is used to excite the sample, and the subsequent fluorescence decay is measured (Becker, 2005; Lakowicz, 2013). For quantitative analysis, the fluorescence exponential decay is fit to an exponential curve. For NAD(P)H and FAD which typically have a short and long lifetime due to conformational changes due to binding, the fluorescence decay curve is fitted to a two-component exponential model, $I\left(t\right)=\alpha_{1}e^{-t/\tau_{1}}+\alpha_{2}e^{-t/\tau_{2}}+C$ , where *I*(*t*) represents the fluorescence intensity as the function of time, *τ₁*, and *τ₂* are the short and long lifetimes respectively, *α₁* and *α₂* are their corresponding fractions, and *C* accounts for background noise. An average fluorescence lifetime can be calculated by the weighted average of short and long lifetimes $\tau_{m}=\alpha_{1}\tau_{1}+\alpha_{2}\tau_{2}$ (Lakowicz, 2013). Although exponential fitting provides quantification of the short and long lifetime values and their relative amplitudes, the fitting procedure is computationally expensive as it requires deconvolution of the fluorescence decay from the instrument response function and an iterative least-squares method to determine the lifetime and fractional values (Becker, 2012). Additionally, a large number of photons must be acquired at every pixel for robust decay analysis, limiting FLIM to small image pixel sizes and long frame integration times.

Alternatively, FLIM data can be analyzed using a phasor transformation. For the phasor transformation, the polar coordinates S and G are computed from the lifetime decay data according to the equations

$$g_{i,j}\left(\omega \right)=\frac{\int_{0}^{T}I_{i,j}\left(t\right)\cos\left(\omega t\right)dt}{\int_{0}^{T}I_{i,j}\left(t\right)dt}$$ (1)

$$s_{i,j}\left(\omega \right)=\frac{\int_{0}^{T}I_{i,j}\left(t\right)\sin\left(\omega t\right)dt}{\int_{0}^{T}I_{i,j}\left(t\right)dt}$$ (2)

where ω=2πf , and *f* is the laser repetition rate (Digman et al., 2008). The S and G coordinates are found for each pixel within a FLIM image and plotted on a polar hemisphere. The phasor plot can be qualitatively assessed as data with a single lifetime will plot on the unit circle with shorter lifetimes on the right. Data with multiple lifetimes will plot within the unit circle along a trajectory of the unit-circle placement of the lifetime values. FLIM analysis with phasors has been used to visualize neural stem cell differentiation (Stringari et al., 2012a; Stringari et al., 2012b) and macrophage phenotypes from NADH lifetime data (Alfonso-Garcia et al., 2016). Phasor analysis is especially beneficial for datasets with 3+ or an unknown number of fluorophore species as it does not require *a priori* knowledge of the number fluorophore lifetimes, and accuracy for multiexponential decays is not dependent on high photon counts as in exponential fitting (Malacrida et al., 2021). Additionally, phasor analysis is advantageous for large datasets due to its fit-free nature and faster analysis times (Malacrida et al., 2021). Phasor analysis is compatible with both time-domain and frequency-domain FLIM instrumentation. However, phasor analysis has been shown to be limited for low signal-to-noise (SNR) FLIM data and is susceptible to instrumentation response errors (Datta et al., 2020).

This study compared the performance of machine learning models that predict cell phenotypes from FLIM features extracted via either phasor analysis or exponential curve fitting. Fluorescence lifetime images of NAD(P)H and FAD images of macrophages chemically polarized to different phenotypes (M0, M1, and M2) were acquired using a two-photon fluorescence lifetime microscope. Quantitative FLIM data was extracted using both a two-component exponential decay curve fit and phasor analysis to compute S and G coordinates. Images were segmented into individual cells. Random forest tree (RFT) algorithms were developed, validated, and compared for classification of the macrophage phenotype for each cell using the FLIM data. Similar analysis were performed for a dataset of metabolically-perturbed MCF7 breast cancer cells to compare the performance of machine learning models to predict cell metabolic states from phasor coordinates or exponential fit values.

## 2 Methods

### 2.1 Cell preparation

THP-1 monocytes (ATCC) were cultured with RPMI-1640 media supplemented with 10% fetal bovine serum (FBS) and 1% penicillin/streptomycin. THP-1 monocytes were seeded on 35 mm glass-bottom imaging dishes with a density of 10^5^ cells/dish and treated with freshly prepared Phorbol 12-myristate 13-acetate (PMA, 50 ng/mL) supplemented with RPMI -1,640 media for 24 h to promote their differentiation into macrophages. Upon 24 h of PMA stimulation, most THP-1 cells become adherent M0 macrophages. For pro-inflammatory (M1) polarization, the macrophages were subsequently treated with Lipopolysaccharides (LPS, 10 ng/mL) and interferon-γ (IFN-γ, 15 ng/mL) for 24 h. M0 macrophages were exposed to interleukin (IL)-4 (20 ng/mL) for anti-inflammatory (M2) polarization. After 24 h of cytokine stimulation, the media was replaced with normal media.

The metabolic perturbation experiment of cancer cells was previously performed by Hu et al., and the procedure summarized here is fully covered in Hu et al. (Hu et al., 2022). MCF7 breast cancer cells were cultured in the Dulbecco’s Modified Eagle’s Medium (DMEM) with glucose (50 mM), pyruvate (2 mM), 1% antibiotic-antimycotic, and 10% fetal bovine serum (FBS). The cells were seeded at a density of 2 × 10^5^ per 35 mm glass-bottom imaging dish 48 h before imaging. The cells were treated with NaCN (4 mM) to inhibit OXPHOS 5 min before imaging. To inhibit glycolysis, 2-Dexoy-D-glucose (2DG, 50 mM) was added to the media 1 h before imaging. Additionally, a glucose depletion group was created by switching the media to no glucose DMEM supplemented with pyruvate (2 mM) 1 h before imaging.

### 2.2 Fluorescence lifetime imaging of NAD(P)H and FAD

Fluorescence lifetime images of NAD(P)H and FAD in cells were captured by a customized multiphoton fluorescence lifetime microscope (Marianas, 3i) equipped with a time-correlated single photon counting (TCSPC) electronics module (SPC-150N, Becker & Hickl). A stage-top incubator (okolab) was set to 37°C, 5% CO_2_, and 85% relative humidity to maintain a physiological environment while imaging. NAD(P)H and FAD in macrophages were excited by a tunable Ti: sapphire femtosecond laser (COHERENT, Chameleon Ultra II) at 750 nm (∼27 mW) and 890 nm (∼35 mW) respectively. The fluorescence lifetime images of NAD(P)H and FAD were obtained sequentially by photomultiplier tube (PMT) detectors (HAMAMATSU, H7422PA-40) and isolated by a 447/60 nm bandpass filter and a 560/88 nm bandpass filter, respectively. For each imaging dish, both NAD(P)H and FAD fluorescence lifetime images were captured in at least five random positions, and each fluorescence lifetime image (256 × 256 pixels) was acquired with a pixel dwell time of 50 μs and 5 frame repeats. The NAD(P)H and FAD fluorescence lifetime images in the cancer cell metabolic inhibitor experiment were previously collected by AJ Walsh and L. Hu with the same imaging system, and the details of autofluorescence imaging are covered in Hu et al. (Hu et al., 2022).

### 2.3 Cell-based fluorescence lifetime analysis

Fluorescence lifetime decays of each NAD(P)H and FAD image were analyzed using SPCImage (Becker & Hickl). The decay curve of each pixel was deconvoluted with the measured instrument response function (IRF), which was obtained by the second harmonic generation of urea crystals, and then fitted into a two-component exponential model. Then, the weight-average fluorescence lifetime was calculated for each pixel with MATLAB to obtain the corresponding *τ*_*m*_ images. The phasor plot analysis was also performed in SPCImage, which converts the time decay of each pixel (*i, j*) to its corresponding phasor coordinates (*g*_*i,j*_(ω), *s*_*i,j*_(ω)) using a laser repetition rate of 80 MHz. As a result, three decay fitting parameters (*α₁*, *τ₁, τ₂*) and two phasor plot coordinates (G, S) were extracted for each pixel in each NAD(P)H and FAD fluorescence lifetime image.

Images were then segmented into individual cells to acquire cell-based fluorescence lifetime features. The cell segmentation process was achieved in CellProfiler using a customized pipeline based on the NAD(P)H intensity images (Walsh and Skala, 2014). As a result, 533–1828 cells were extracted for each group with 3 technical replicates (Supplementary Table S1). Finally, image processing was performed using a customized script in MATLAB, and the pixel-averaged values for eight NAD(P)H and FAD fluorescence decay fitting features including NAD(P)H *τ₁*, NAD(P)H *τ₂*, NAD(P)H *α₁*, NAD(P)H *τ*_*m*_, FAD *τ₁*, FAD *τ₂*, FAD *α₁*, FAD *τ*_*m*_, and four phasor plotting features including NAD(P)H G, NAD(P)H S, FAD G, and FAD S, were acquired for each cell.

### 2.4 Statistical analysis and ML model development

The statistical analysis and machine learning (ML) model development were performed using customized scripts in Rstudio. A two-sided t-test was applied to compare different autofluorescence lifetime features across various cell groups, and a *p* value of 0.05 was set as the threshold to identify significance. The Uniform Manifold Approximate and Projection (UMAP) was used to reduce the data dimensions to visualize clusters within the autofluorescence imaging datasets. Random forest tree (RFT) machine learning algorithms were trained to classify macrophage phenotypes or cancer cell metabolism based on the features of lifetime decay fitting and phasor coordinates, respectively. Each model was evaluated using 5-fold cross-validation, which trained the model with 80% of the dataset and tested the model with the remaining 20% of the dataset on five iterations. Furthermore, the AUC (area under the curve) of the receiver operation characteristic (ROC) curves of the models evaluated on the test datasets were used to evaluate the performance of the models. A two-sided t-test was applied to compare the average accuracy and ROC AUC of the 5-fold repeated models across different classifiers. The relative contributions of each feature in each classifier were obtained by the mean decrease gini from the RFT.

## 3 Results

### 3.1 Phasor analysis and decay curve fitting for analysis of autofluorescence lifetime images of macrophage phenotypes

**FIGURE 1:**
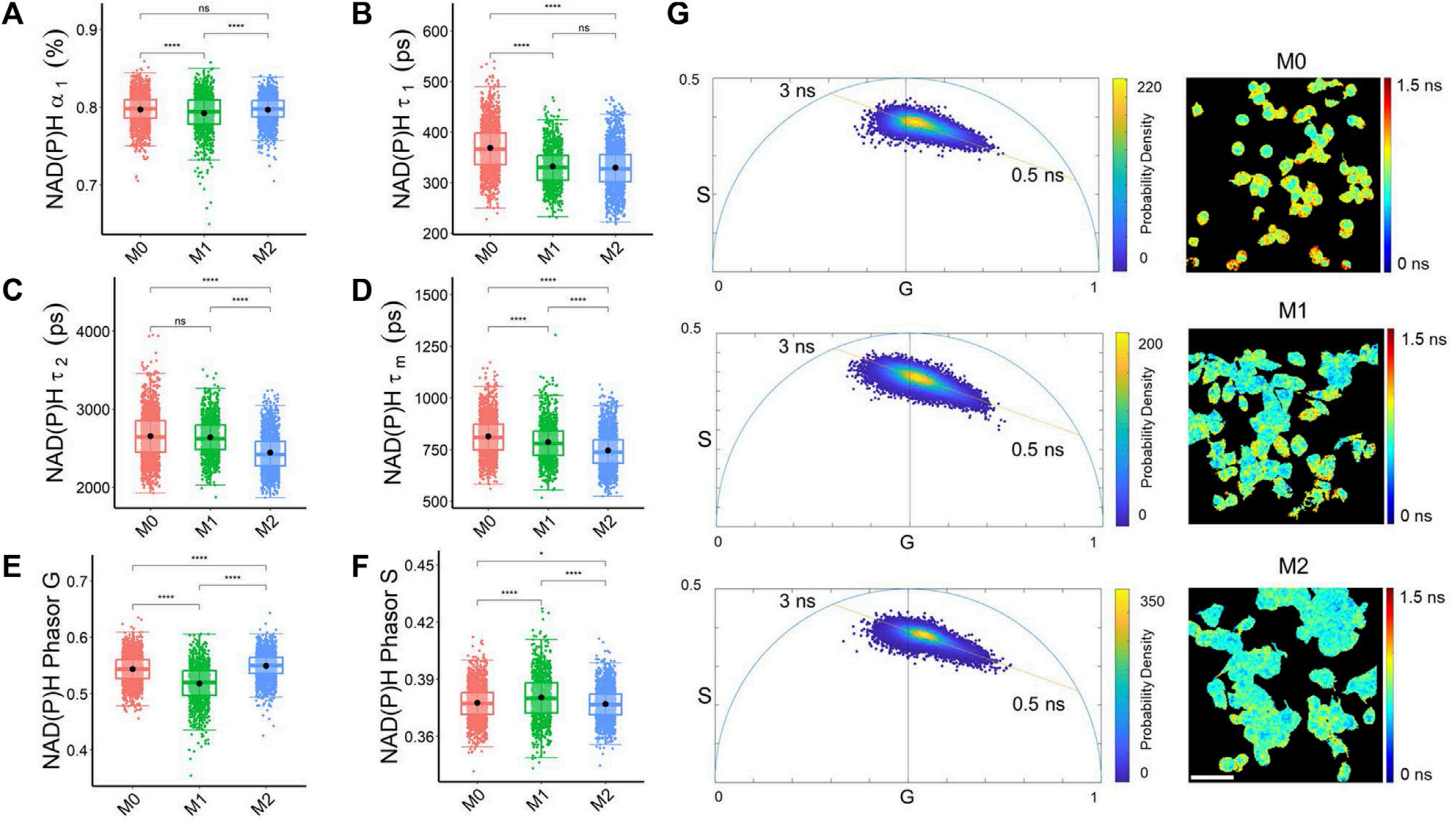
Decay curve fitting and phasor analysis resolve metabolic variations among macrophage phenotypes. **(A)** NAD(P)H free fraction (*α₁*) **(B)** Free NAD(P)H lifetime (*τ₁*) **(C)** bound NAD(P)H lifetime (*τ₂*) **(D)** Average NAD(P)H lifetime (*τ*_*m*_) **(E)** NAD(P)H phasor G **(F)** NAD(P)H phasor S reveal differences in the quantified NAD(P)H fluorescence lifetimes of M0, M1, and M2 macrophages. *p < 0.05, **p < 0.01, ****p < 0.0001 for two-sided Student’s t-test. Each data point is the pixel-averaged value for a single cell, n = 1828 cells for M0, n = 1,074 cells for M1, n = 1706 cells for M2 **(G)** Representative NAD(P)H mean lifetime image (*τ*_*m*_) and corresponding phasor plot for M0 (top), M1 (middle), and M2 (bottom) macrophages. The color in the phasor plot represents the estimated probability density. Scale bar = 60 μm. Each data point on the phasor plot corresponds to a single pixel in the FLIM image.

Both biexponential lifetime decay curve fitting and phasor analysis revealed different characteristics of NAD(P)H and FAD fluorescence lifetime across the macrophage phenotypes. M1 macrophages had a lower fraction of free NAD(P)H (*α₁*) than M0 and M2 macrophages, which was also captured by the phasor analysis with an increased NAD(P)H phasor S value and lower NAD(P)H phasor G value in M1 macrophages compared to M0 and M2 macrophages (Figures 1A, E, F). The fraction of free NAD(P)H (*α₁*) represents the pool of unbound NAD(P)H molecules available for metabolic reactions, and changes in this fraction can suggest alterations in the utilization of NAD(P)H in different macrophages related to a shift in metabolic activities. Both M1 and M2 macrophages had a shorter free NAD(P)H lifetime (*τ₁*), and M2 macrophages had a shorter bound NAD(P)H lifetime (*τ₂*) than M0 and M1 macrophages (Figures 1B, C). The lifetimes of bound NAD(P)H (*τ₂*) reflect the dynamics of NAD(P)H interactions with specific proteins in distinct metabolic pathways. Variations in the free and bound lifetimes between macrophages indicate changes in the kinetics of NAD(P)H binding and release involved in metabolic pathways. These variations of NAD(P)H lifetime components led to the shortest mean NAD(P)H lifetimes (*τ*_*m*_) in M2 macrophages (Figure 1D).

In the representative NAD(P)H phasor plot images, most pixels clustered along a line between the lifetimes of free NAD(P)H (0.5 ns) and bound NAD(P)H (∼3 ns) (Figure 1G). M1 macrophages had a higher fraction of bound FAD, longer bound FAD lifetime, and shorter free FAD lifetime than M0 and M2 macrophages (Supplementary Figures S1A–C). These lifetime variations resulted in a shorter mean FAD lifetime than M0 and M2 macrophages (Supplementary Figure S1D). Alterations in metabolic pathways, such as the tricarboxylic acid (TCA) cycle, electron transport chain, and fatty acid oxidation, can impact the availability and utilization of FAD, consequently resulting in variations in FAD lifetime. The phasor plot analysis of FAD fluorescence lifetime showed that M1 macrophages had a higher FAD phasor G value and lower FAD S value than the M0, and M2 macrophages (Supplementary Figures S1E, F; Supplementary Table S2).

### 3.2 RFT models predict metabolic phenotypes of macrophages from FLIM data

**FIGURE 2:**
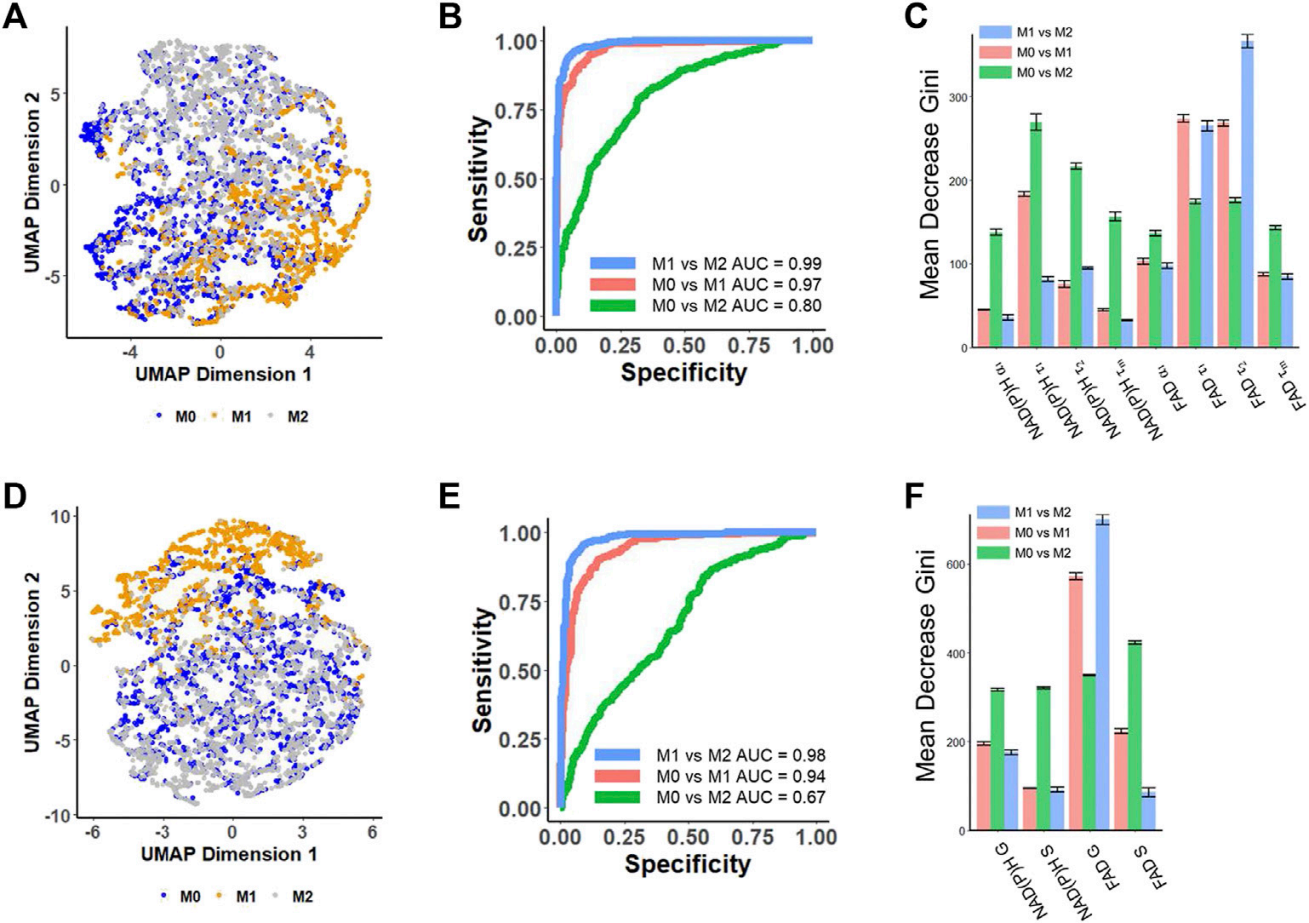
Prediction of metabolic phenotypes of macrophages with autofluorescence lifetime features [**(A–C)** use features from decay fitting, **(D–F)** use features from phasor plotting]. **(A)** UMAP of NAD(P)H and FAD autofluorescence lifetime features from decay fitting in M0, M1, and M2 macrophages. Each dot corresponds to a single cell, blue corresponds to M0 macrophages, yellow corresponds to M1 macrophages, and gray corresponds to M2 macrophages. **(B)** ROC curves of the test data for RFT machine-learning models developed based on decay fitting features for the classification of different macrophage phenotypes. **(C)** Feature importance (mean decrease gini) of curve-fitting features within RFT classifiers for predicting macrophage phenotypes. **(D)** UMAP of NAD(P)H and FAD autofluorescence lifetime features from phasor analysis in M0, M1, and M2 macrophages. Each dot corresponds to a single cell, blue corresponds to M0 macrophages, yellow corresponds to M1 macrophages, and gray corresponds to M2. **(E)** ROC curves of the test data for RFT machine-learning model developed based on phasor analysis features for the classification of different macrophage phenotypes. **(F)** Feature importance (mean decrease gini) of phasor plot features within RFT classifiers for predicting macrophage phenotypes.

UMAP was used to visualize separation of cell phenotypes by compressing the variance in the multivariate lifetime datasets into two dimensions. The UMAP generated from eight different NAD(P)H and FAD lifetime decay curve fitting features (NAD(P)H: *α₁*, *τ₁*, *τ₂*, *τ*_*m*_; FAD: *α₁*, *τ₁*, *τ₂*, *τ*_*m*_) showed that M1 macrophages are predominantly positioned at the bottom right, exhibiting a subtle separation from the other two macrophage phenotypes (Figure 2A). The separation observed on the UMAP based on lifetime decay suggests the potential for classifying macrophage phenotypes. However, the M0 macrophage cluster overlapped with M2 macrophages (Figure 2A).

Random forest tree models were generated for pairwise classification of macrophage phenotype. When applying random forest tree algorithms to predict macrophage phenotypes, the model achieved a mean prediction accuracy of 94.6% and an AUC value of 0.99 for classifying M1 macrophages *versus* M2 macrophages (Figure 2B; Supplementary Table S2), and the free FAD lifetime (*τ₂*) and bound FAD lifetime (*τ₁*) were the highest weighted features for this classification (Figure 2C). Moreover, a RFT model could also discriminate between M0 and M1 macrophages with an AUC of 0.97 and an average accuracy of 92.6% (Figure 2B; Supplementary Table S2). In this model, the bound FAD lifetime (*τ₁*), free FAD lifetime (*τ₂*), and free NAD(P)H lifetime (*τ₁*) contributed most to the classification (Figure 2C). The strong contributions of free and bound FAD lifetimes (*τ₁*, *τ₂*) in both classifiers highlight the importance of FAD metabolism and dynamics in distinguishing between macrophage phenotypes. However, the RFT trained with NAD(P)H and FAD features of decay fitting did not provide high performance in classifying M0 and M2 macrophage phenotypes (Figure 2B; Supplementary Table S2). A RFT for classifying M0-M2 macrophages had an average accuracy of 71.1% and an AUC value of 0.80 (Figure 2B; Supplementary Table S2), with the free NAD(P)H lifetime (*τ₁*) and bound NAD(P)H lifetime (*τ*_*2*_) contributing significantly to this classification (Figure 2C).

The phasor coordinates of NAD(P)H and FAD FLIM images also classify macrophage phenotypes. The UMAP visualization based on four different NAD(P)H and FAD phasor plot features (NAD(P)H: G, S; FAD: G, S) showed that the M1 macrophages are located at the top, and exhibit clustering separate from the M0 and M2 macrophages (Figure 2D). Applying these features to predict macrophage phenotypes using a RFT machine learning algorithm achieved an AUC of 0.98, and an average accuracy of 0.924 for classifying M1 *versus* M2 macrophage phenotypes (Figure 2E; Supplementary Table S3). The phasor features achieved a classification of M0 *versus* M1 macrophage phenotypes with an AUC of 0.94 and an average accuracy of 89.7% (Figure 2E; Supplementary Table S3). These results imply that phasor coordinates of autofluorescence lifetime images are effective at classifying the macrophage phenotypes accurately. FAD G was the most important feature in both of these two classifiers (Figure 2F). The autofluorescence phasor analysis features did not discriminate M0 *versus* M2 macrophage phenotypes with high accuracy. The RFT model to predict M0 from M2 macrophages had an AUC of 0.67 and an average accuracy of 64.1% (Figure 2E; Supplementary Table S3).

To statistically compare the performance of the different classifiers in discriminating macrophage phenotypes, the average accuracy and ROC AUC of the 5-fold cross-validation were compared using a two-sided t-test. The RFT models trained with decay fitting features demonstrated a significantly higher accuracy (*p* < 0.001) compared to the models trained with phasor features when classifying M0 *versus* M1 (approximately 3% improvement) and M0 *versus* M2 (approximately 7% improvement) (Supplementary Tables S2, S3, S8). Furthermore, the combination of both decay fitting and phasor features further improved the classification accuracy significantly (*p* < 0.01) for discriminating M0 and M1, as well as M0 and M2 macrophages (Supplementary Tables S4, S8).

### 3.3 Phasor analysis identifies NAD(P)H and FAD lifetime distributions in cancer cells exposed to metabolic perturbations

**FIGURE 3:**
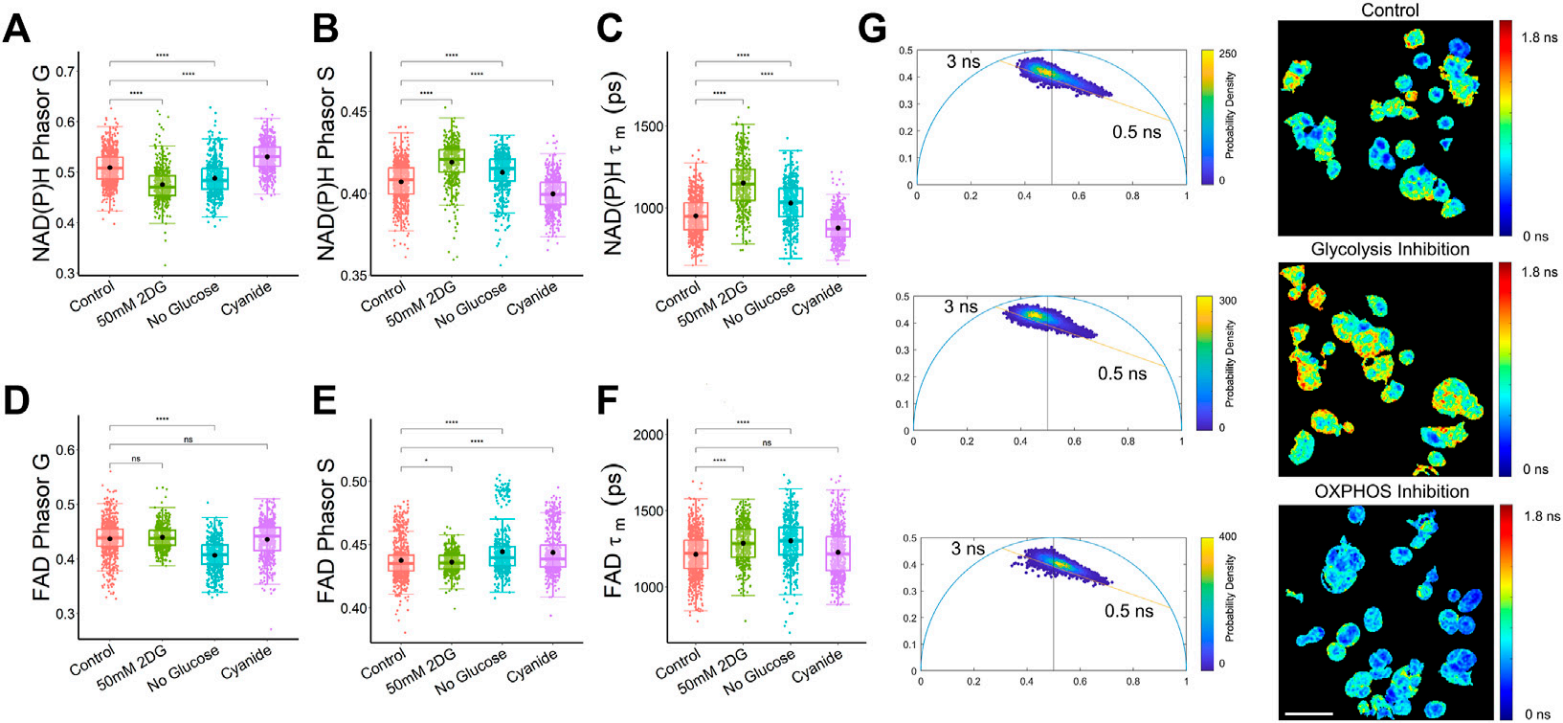
Phasor analysis and decay curve fitting resolve metabolic states of MCF7 cells. **(A)** NAD(P)H phasor G **(B)** NAD(P)H phasor S **(C)** NAD(P)H *τ*_*m*_ **(D)** FAD Phasor G **(E)** FAD phasor S **(F)** FAD *τ*_*m*_ values reveal differences in the NAD(P)H and FAD fluorescence lifetimes of MCF7 cells exposed to control media, media with 2DG at 50 mM, media without glucose, and media with cyanide. *p < 0.05, ****p < 0.0001 for two-sided Student’s t-test. Each data point is the pixel-averaged value for a single cell, n = 472–839 cells per group. **(G)** Representative NAD(P)H mean lifetime image (*τ*_*m*_) and corresponding phasor plot. The color in the phasor plot represents the estimated probability density. Scale bar = 60 μm.

The phasor analysis from a dataset of MCF7 cells exposed to metabolic conditions to stimulate and inhibit glycolysis and oxidative phosphorylation showed that the mean NAD(P)H phasor components G and S were statistically different among control, 2DG-treated, and cyanide-treated cells (Figures 3A, B). The FAD phasor components were inconclusive, with the mean G coordinate of the control MCF7 cells not statistically different from the cyanide (OXPHOS inhibition) or the glycolysis inhibition groups (Figure 3D). The MCF7 cells treated with cyanide for OXPHOS inhibition had a higher mean G component and a lower S component for NAD(P)H compared to the control group (Figures 3A, B). While the FAD mean lifetime (*τ*_*m*_) showed a statistically significant difference between control cancer cells and both the glycolysis inhibition groups, 50 mM 2DG and no glucose, only the no glucose group was statistically different from control cells for both FAD Phasor G and S values (Figures 3D–F), potentially indicating that the exponential decay fitting method was more sensitive for FAD differences in cancer cells with glycolysis inhibition (Figure 3F). The NAD(P)H phasor plots shown in Figure 3G allow visualization of shifts in the NAD(P)H lifetime of MCF7 cells along the 0.5–3 ns axis due to metabolic perturbations without using a biexponential curve fitting. The cells with OXPHOS inhibition exhibited a shift towards more free NAD(P)H with a shorter fluorescence lifetime compared to the control and glycolysis-inhibited group (Figure 3G). Cyanide inhibits the electron transport chain complex IV (Marziaz et al., 2013), leading to this increase in free NADH, as observed previously in MCF10A breast cancer cells (Drozdowicz-Tomsia et al., 2014). For the glycolysis inhibition group, the phasor plot allows visualization of the shift towards bound NADH, which can be attributed to the fact that free NADH is produced during glycolysis (Hu et al., 2022).

### 3.4 Autofluorescence lifetime features predict metabolic phenotypes of cancer cells

**FIGURE 4:**
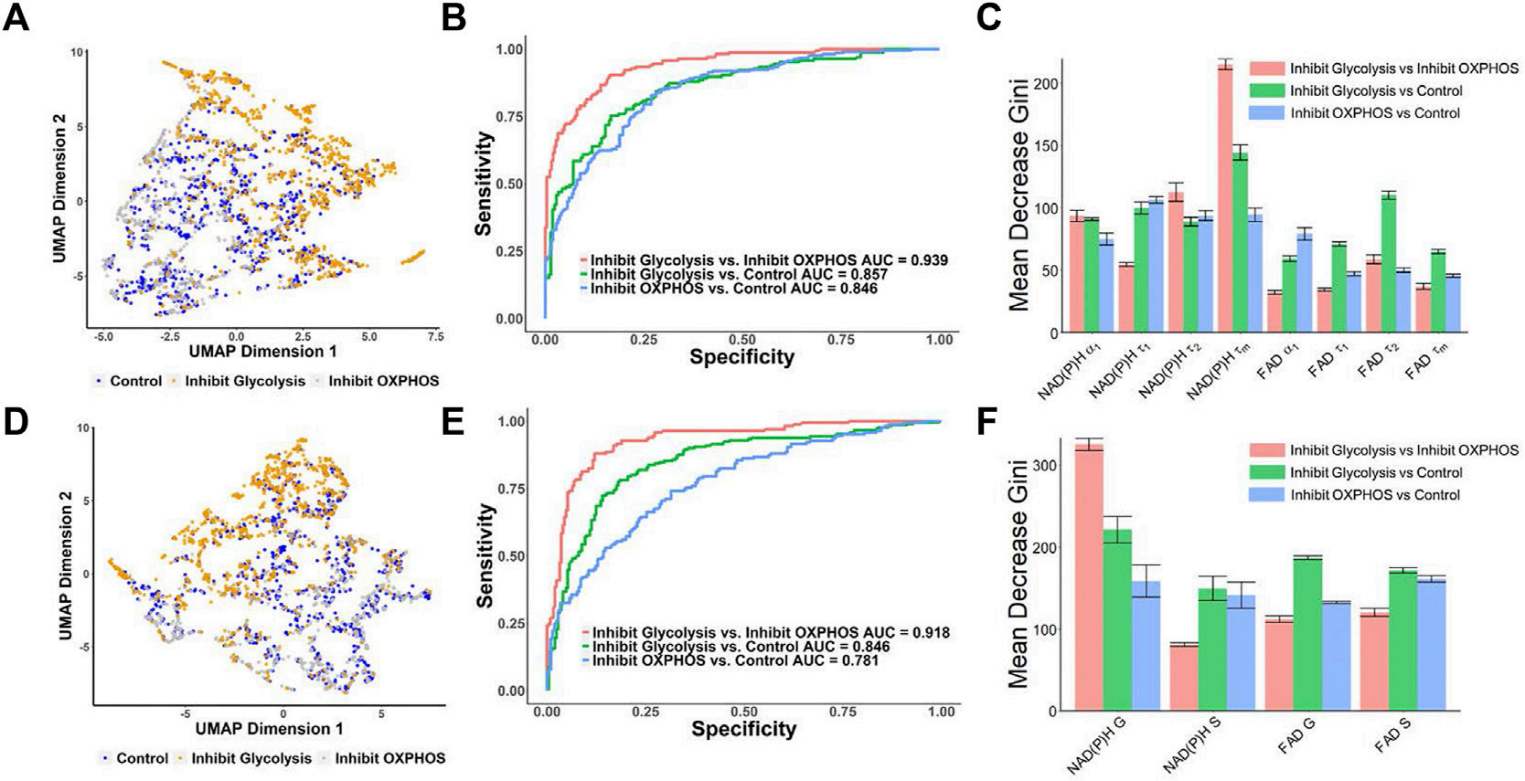
Prediction of the metabolic states of MCF7 cells from autofluorescence lifetime features [**(A–C)** use features from decay fitting, **(D–F)** use features from phasor plotting]. **(A)** UMAP of NAD(P)H and FAD autofluorescence lifetime features from decay fitting for control, 2DG and no glucose treatment (glycolysis inhibition) and cyanide groups (OXPHOS inhibition). Each dot corresponds to a single cell, blue corresponds to the control group, orange corresponds to the 2DG and no glucose treatment (glycolysis inhibition), and gray corresponds to the cells treated with cyanide (OXPHOS inhibition). **(B)** ROC curves of test data predicted from RFT machine-learning model using decay fitting features for the different metabolic states of the cancer cells. **(C)** Feature importance (mean decrease gini) of curve-fitting features within RFT classifiers for predicting metabolic states of cancer cells. **(D)** UMAP of NAD(P)H and FAD autofluorescence S and G phasor components for classification of different metabolic states of cancer cells. Each dot corresponds to a single cell, blue corresponds to the control group, orange corresponds to the 2DG and no glucose treatment (glycolysis inhibition), and gray corresponds to the group treated with cyanide (OXPHOS inhibition). **(E)** ROC curves of test data predicted from RFT machine-learning model using phasor analysis features for the different metabolic states of the cancer cells. **(F)** Feature importance (mean decrease gini) of phasor plot features within different RFT classifiers for predicting metabolic states of cancer cells.

UMAP visualization of MCF7 metabolic perturbation dataset from the FLIM decay fitting components (NAD(P)H: *α₁*, *τ₁*, *τ₂*, *τ*_*m*_; FAD: *α₁*, *τ₁*, *τ₂*, *τ*_*m*_) showed there was some separation between the glycolysis inhibition group clusters from the control and OXPHOS inhibition groups (Figure 4A). Similarly, a UMAP generated from the phasor components (NAD(P)H: G and S; FAD: G and S) showed similar patterns to the UMAP generated from the decay fitting variables, where a separation between the glycolysis inhibition group and the other two groups (control and OXPHOS inhibition) is apparent (Figure 4D) indicating that there is a potential for classifying the metabolic states in breast cancer cells. To test this, RFT machine learning models were created and compared for classifying MCF7 cell metabolism phenotype from NAD(P)H and FAD fluorescence lifetime metrics. Using lifetime features from decay fitting, the RFT machine learning algorithm provided an overall higher AUC compared to using only phasor analysis components S and G to differentiate between the control, glycolysis inhibition, and OXPHOS inhibition groups (Figures 4B, E). The model built from exponential decay fitting features had an AUC of 0.857 and an average accuracy of 78.5% for the classification of the glycolysis inhibition group from the control group (Figure 4B; Supplementary Table S5), whereas the model for the same comparison built from phasor coordinates had an AUC of 0.846, and an average accuracy of 75.5% (Figure 4E; Supplementary Table S6). For distinguishing between the glycolysis inhibition and OXPHOS inhibition group, the RFT algorithms achieved an AUC of 0.939 and an average accuracy of 86.3% for the exponential curve fitting data (Figure 4B; Supplementary Table S5). Comparatively, an AUC of 0.918 and an average accuracy of 87.7% were achieved for the glycolysis *versus* OXPHOS RFT model created from the phasor coordinates (Figure 4E; Supplementary Table S6). Neither of the models trained with the features of decay-fitting or phasor analysis discriminated the OXPHOS inhibition and control group achieved an ROC AUC value greater than 0.9 (Figures 4B, E).

NAD(P)H mean lifetimes ( $\tau_{m})$ contributed the most for differentiating between the glycolysis inhibition and OXPHOS inhibition groups as well as between the glycolysis inhibition group and the control for the lifetime components ML model (Figure 4C). The NAD(P)H free ( $\tau_{1})$ , bound ( $\tau_{2})$ , and mean lifetimes $\left(\tau_{m}\right)$ contributed to the classification of OXPHOS inhibition and the control group. For the phasor components, NAD(P)H G component was found to be the most critical feature for distinguishing between the control and glycolysis inhibition groups and the glycolysis inhibition and OXPHOS inhibition (Figure 4F). Furthermore, all NAD(P)H and FAD phasor components were important factors in classifying the control and OXPHOS inhibition groups (Figure 4F). The combination of decay fitting and phasor features did not significantly improve the prediction accuracies of glycolysis inhibition *versus* OXPHOS inhibition (Supplementary Tables S7, S8).

## 4 Discussion

Autofluorescence lifetime imaging of endogenous fluorophores NAD(P)H and FAD provides a label-free, nondestructive method for quantifying metabolic variations in live cells, allowing for the classification of different phenotypes of cancer cells, stem cells, and immune cells using machine learning algorithms (Liu et al., 2018; Qian et al., 2021; Walsh et al., 2021; Hu and Walsh, 2022; Neto et al., 2022). Time-domain fluorescence lifetime data can be analyzed using both curve fitting and phasor analysis to extract lifetime values and phasor coordinates, respectively. Exponential curve fitting analysis of NAD(P)H and FAD lifetime quantifies the fraction of free and bound molecules, along with their corresponding fluorescence lifetimes. However, exponential decay fitting requires a number of assumptions about the dataset, such as the number of lifetime components and shifts in instrument response function, that necessitate domain expertise. Moreover, the deconvolution process before fitting can be challenging and a higher number of photons are required for an accurate fit, which limits the applicability of exponential fitting in cases with low autofluorescence and high background noise (Becker, 2012; Datta et al., 2020).

Alternatively, phasor analysis provides a fit-free visualization of lifetime estimates, allowing for faster lifetime analysis with fewer computational tasks and does not require assumptions of the decay parameters (Ranjit et al., 2018). Because pixels with similar fluorescence lifetimes cluster on the polar plot, phasor analysis allows rapid visualization and identification of image regions with either similar or different fluorescence lifetimes (Ranjit et al., 2018). Even though phasor analysis does not directly quantitate lifetime values or component weights, phasor analysis is compatible with both time and frequency-domain lifetime imaging systems and can overcome deconvolution and exponential decay fitting limitations (Digman et al., 2008; Ranjit et al., 2018; Malacrida et al., 2021). Given that phasor plot analysis and curve fitting analysis present lifetime variations in different formats, it is important to compare the effectiveness of these two methods to extract fluorescence lifetime features for subsequent discriminating algorithms, such as machine learning models for cell classification. In this paper, we employ RFT ML models to evaluate and compare the performance of features derived from decay fitting analysis and phasor plot analysis in predicting metabolic phenotypes of cancer and immune cells.

Under normal conditions, pro-inflammatory macrophages (M1) depend on glycolysis, whereas monocytes (M0) and anti-inflammatory macrophages (M2) typically use oxidative phosphorylation (OXPHOS) to maintain their metabolic requirements (Galvan-Pena and O'Neill, 2014; Kelly and O'Neill, 2015; Verdeguer and Aouadi, 2017). The metabolic variations in macrophages can be visualized through NAD(P)H and FAD fluorescence lifetime imaging, and metabolic phenotypes can be distinguished by machine learning algorithms using autofluorescence features (Figures 1, 2) (Neto et al., 2022). Although previous studies have observed an increased fraction of bound NAD(P)H in M2 macrophages and an increased free fraction of NAD(P)H in M1 macrophages *in vitro* (Alfonso-Garcia et al., 2016; Heaster et al., 2020), *in vivo* studies of macrophages in zebrafish demonstrate a decreased mean NAD(P)H lifetime, due to decreased short and long NAD(P)H lifetimes, and an increased NAD(P)H *α*_*1*_ within the macrophages in tail wounds (Miskolci et al., 2022). The inconsistencies observed in NAD(P)H lifetimes among various macrophage phenotypes may be attributed to differences in macrophage origins, polarization protocols, and intra-group cellular heterogeneity. Metabolic differences, particularly in lipid and amino acid metabolism, have been identified between human THP-1 cells and rat bone marrow-derived macrophages, which can impact NAD(P)H and FAD fluorescence lifetimes (Gross et al., 2014; Namgaladze and Brune, 2014; Batista-Gonzalez et al., 2019). Furthermore, perturbations in NAD(P)H and FAD fluorescence lifetime in macrophages have been observed in various circumstances, including wound healing (Miskolci et al., 2022), and tumor association (Szulczewski et al., 2016; Heaster et al., 2020; Heaster et al., 2021), indicating the existence of metabolic heterogeneity within macrophage populations.

Machine learning RFT models achieved similar accuracies with ROC AUC values > 0.94 for the classification of M1 from M2 macrophages and the classification of M0 from M1 macrophages for models built from autofluorescence lifetime metrics quantified either via decay fitting or phasor analysis (Figures 2B, E). Similar results have been previously reported for a RFT model that predicts M1 and M2 phenotype of human macrophages polarized and treated with FCCP (ROC AUC = 0.944) (Neto et al., 2022). Although significant differences in NAD(P)H lifetimes (*τ*_*1*_, *τ*_*2*_, *τ*_*m*_), were observed between M0 and M2 macrophages (Figures 1B–D), the random forest tree (RFT) models designed to distinguish between these subsets did not achieve a high accuracy (∼71%) (Supplementary Table S2), suggesting that the differences in lifetime features may not be sufficient for effective classification. Both M0 and M2 macrophages rely on oxidative phosphorylation (OXPHOS) for energy production, suggesting a biological explanation for overlapping metabolic characteristics of lifetime features that prevent robust classification of M0 and M2 macrophages from autofluorescence lifetime-based models (Ravi et al., 2014).

For the M0 *versus* M2 models, better accuracy (*p* < 0.0001) was achieved for the RFT model that used FLIM features from the fitted decay than the model built from the phasor coordinates (ROC AUC of 0.8 *versus* 0.67; Figures 2B, E; Supplementary Table S8). It is noteworthy that FAD fluorescence lifetime features contributed more to the M1 *versus* M2 models than the NAD(P)H features (Figures 2E, F), which has not been previously reported. FAD fluorescence lifetimes may be associated with alterations in mitochondrial function and metabolism, suggesting that mitochondria-dependent metabolism drives phenotypic changes between M1 and M2 macrophages.

Similarly to activated macrophages, cancer cells exhibit increased aerobic glycolysis to support proliferation even when oxygen is present, a phenomenon known as the Warburg effect (Warburg, 1956). Autofluorescence lifetime imaging combined with either exponential decay fitting or phasor analysis allows analysis of metabolic phenotypes of cancer cells from FLIM data (Periasamy et al., 2012; Walsh et al., 2014; Alam et al., 2017; Trinh et al., 2017; Pham et al., 2021). The phasor plots showed that glycolysis inhibition within MCF7 cells led to a shift towards more bound NADH, while the OXPHOS inhibition stimulated a shift toward more free NADH (Figure 3G). These results are consistent with previously reported NAD(P)H lifetime phasor shifts of MCF7 cells treated with caffeine enhancement of cisplatin, a chemotherapy drug that alters the metabolic state of a cancer cell and induces mitochondrial apoptosis (Pascua et al., 2020). The MCF7 cells trended toward OXPHOS when treated with a combination of caffeine and cisplatin, representative of a lower amount of free NAD(P)H (Pascua et al., 2020). Additionally, a significant difference in the FAD phasor components was observed between the cells with glucose starvation and the control cells (Figures 3D, E), which has been shown in previous studies for lifetime components as well (Supplementary Figure S3) (Hu et al., 2022).

Biexponential fluorescence decay curve fitting analysis of NAD(P)H and FAD fluorescence lifetimes resolve metabolic variations of breast cancer cells and further allows for the classification of metabolic phenotypes at a single-cell level using machine learning algorithms (Hu et al., 2022). We expanded on this work by comparing the performance of the RFT machine learning models trained on phasor features, curve fitting features, and combined features from both analysis approaches. Using the phasor features of autofluorescence lifetime as inputs to the RFT model proved not as effective as the curve-fitting features in discriminating metabolic phenotypes of cancer cells. Although the model trained with phasor features distinguished between glycolysis inhibition and OXPHOS inhibition groups with an AUC of 0.918, the model was only marginally able to accurately differentiate the control group from the glycolysis inhibition groups (AUC of 0.846) and control from the OXPHOS inhibition group (AUC of 0.781; Figure 4E). In contrast, the model trained with curve fitting features had AUC values ∼85% (Figure 4B) for predictions of control *versus* OXPHOS and control *versus* glycolysis. The increased number of features provided by bi-exponential decay fitting could contribute to the slight increases in average accuracies of the RFT models built from fitting features rather than phasor coordinates. The NAD(P)H G coordinate contributed significantly to the classification of cells with glycolysis inhibition and cells with OXPHOS inhibition (Figure 4C), and the ML model trained with lifetime features (Figure 4F) was generally more dependent on NAD(P)H *τ*_*m*_, suggesting that NAD(P)H lifetime is the most informative metric for characterizing cellular metabolic states.

Both biexponential decay fitting and phasor analysis have advantages and limitations for the analysis of NAD(P)H and FAD FLIM data. In a direct comparison of FLIM features for machine learning classification, RFT models built from biexponential decay fitting features and phasor coordinate yielded similar accuracies for distinguishing between groups with large metabolic differences (M1 *versus* M2 Figure 2; OXPHOS inhibited *versus* glycolysis inhibited cancer cells; Figure 4). However, the accuracy of the phasor-based models decreased relative to the matched models constructed from exponential fit parameters for groups with smaller differences in metabolism (M0 *versus* M2 Figure 2.; OXPHOS inhibited *versus* control cancer cells; Figure 4). However, curve-fitting is computationally intensive and can be prone to inaccuracies caused by the variations of photon numbers. In contrast, phasor coordinates can be quickly calculated without requiring detailed decay curve information or fitting, making it useful for large time-domain or frequency-domain lifetime datasets.

It is important to note that the classifiers developed here for cancer cells and macrophages may potentially transfer to classify additional cell types by metabolic phenotypes if the metabolism and autofluorescence lifetimes of the new cell types are similar to those of cancer cells and macrophages. Likewise new classifiers may be developed to classify mixed populations of cells, provided that the cells have different sufficiently different metabolic phenotypes and autofluorescence lifetimes. However, the autofluorescence lifetime features alone may not be sufficient to distinguish between cell types if 2 cell types share similar metabolic states, as was observed for M0 and M2 macrophages (Figure 2). To overcome this limitation, additional cellular features, such as cell size, morphology, and texture, derived from autofluorescence lifetime images could be included in classifier models in addition to the lifetime properties. These morphological features can be obtained through appropriate imaging preprocessing techniques and may provide additional information for differentiation among multiple cell types.

## 5 Conclusion

In summary, this paper compares curve fitting and phasor analysis approaches for analyzing time-domain NAD(P)H and FAD FLIM data of cancer cells and macrophages across metabolic and phenotypic states. Quantified phasor coordinates identify differences in NAD(P)H and FAD lifetimes of metabolically-perturbed cancer cells and M1 and M2 macrophages. The phasor coordinates were used in machine learning algorithms to predict metabolic phenotypes of cancer cells and macrophage phenotypes. Comparable accuracy was obtained for RFT models using either exponential decay features or phasor coordinates for the prediction of OXPHOS from glycolytic cancer cells and M1 from M2 macrophages. RFT models trained on biexponential lifetime features achieved slightly higher accuracies (∼2–3%) than data-matched phasor-based models for discriminating cancer cell metabolism and macrophage phenotypes.

## Data Availability

The original contributions presented in the study are included in the article/Supplementary Material, further inquiries can be directed to the corresponding author.
