# Supplementary material for: Comparison of phasor analysis and biexponential decay curve fitting of autofluorescence lifetime imaging data for machine learning prediction of cellular phenotypes
Source: Front Bioinform. 2023 Jun 29;3:1210157. doi: 10.3389/fbinf.2023.1210157 (PMC10342207; doi:10.3389/fbinf.2023.1210157)
Supplement: Supplementary file 2 [file DataSheet1.pdf]

Supplementary Material

Comparison of phasor analysis and biexponential decay curve fitting of autofluorescence lifetime imaging data for machine learning prediction of cellular phenotypes

Linghao Hu[1†], Blanche ter Hofstede[1†], Dhavan Sharma[1], Feng Zhao[1], Alex J. Walsh1[1*]

[†]These authors contributed equally to this work and share first authorship

*** Correspondence:** Alex J. Walsh: walshaj@tamu.edu

1 Supplementary Data

The curve-fitting fluorescence lifetime values and phasor plot components of each cell were presented in the Excel file. The MATLAB and R codes for lifetime analysis can be found at https://github.com/walshlab/PhasorML

2 Supplementary Figures and Tables.

2.1 Supplementary Figures

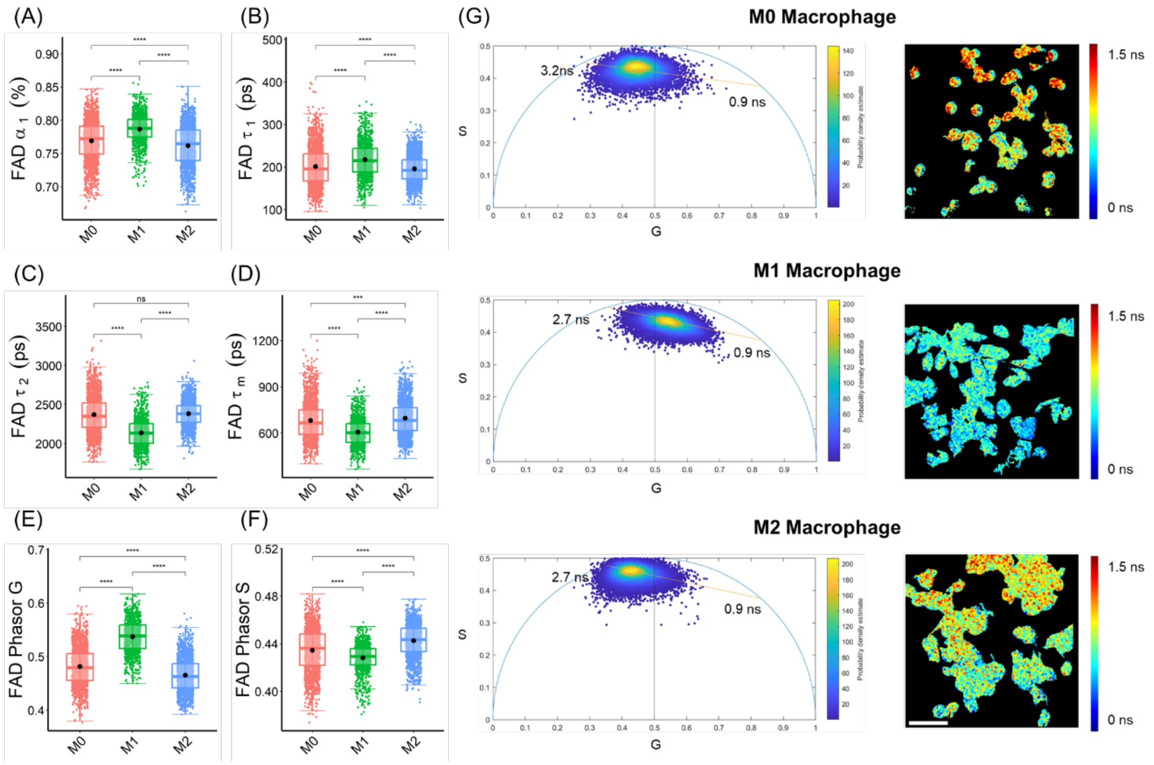

Supplementary Figure S1. Decay curve fitting and phasor analysis resolve metabolic variations among macrophage phenotypes. (A) FAD bound fraction (α_1) (B) Bound FAD lifetime (τ_1) (C) Free FAD lifetime (τ_2) (D) Average FAD lifetime (τ_m) (E) FAD phasor G (F) FAD phasor S reveal differences in the quantified FAD fluorescence lifetimes of M0, M1, and M2 macrophages. ***P < 0.001, ****P < 0.0001 for two-sided student's t-test. Each data point is the pixel-averaged value for a single cell, n=1828 cells for M0, n=1074 cells for M1, n=1706 cells for M2 (G) Representative FAD mean lifetime image (τ_m) and corresponding phasor plot for M0 (top), M1 (middle), and M2 (bottom) macrophages. The color in the phasor plot represents the estimated probability density. Scale bar = 60 µm. Each data point on the phasor plot corresponds to a single pixel in the FLIM image.

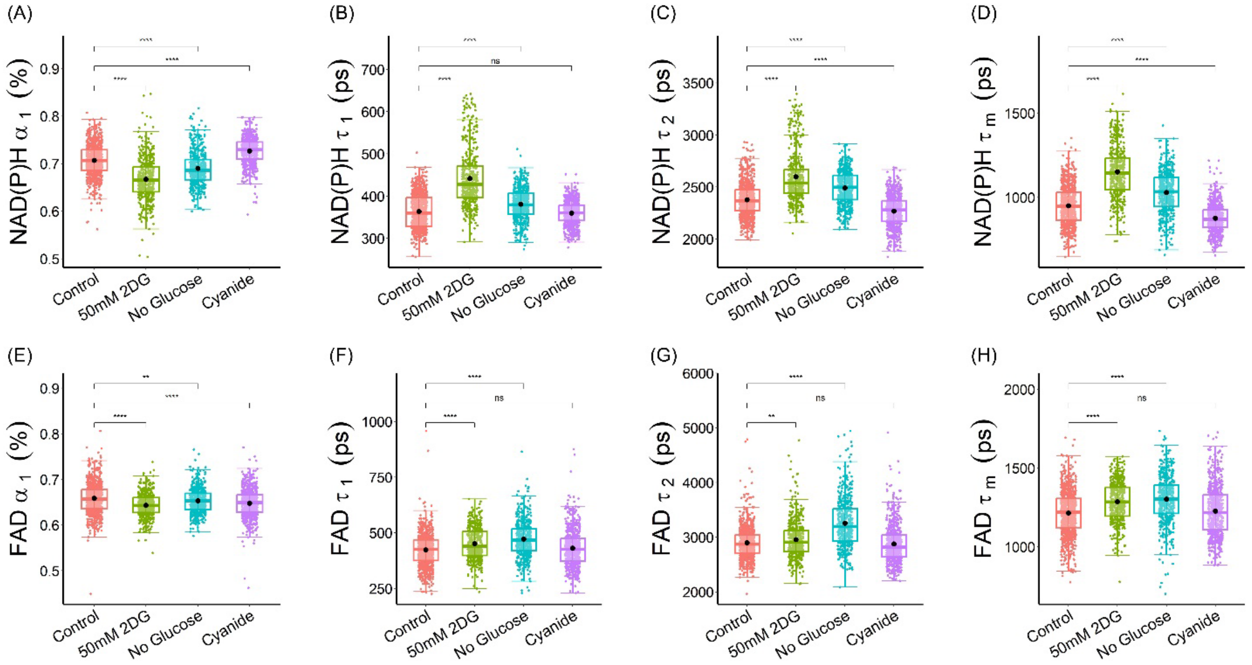

Supplementary Figure S3. Decay curve fitting analysis of NAD(P)H and FAD lifetime images resolve metabolic variations among cancer cells. **(A)** NAD(P)H free fraction (α_1), **(B)** Free NAD(P)H lifetime (τ_1), **(C)** bound NAD(P)H lifetime (τ_2), **(D)** Average NAD(P)H lifetime (τ_m), **(E)** FAD free fraction (α_1), **(F)** Bound FAD lifetime (τ_1), **(G)** Free FAD lifetime (τ_2), **(H)** Average FAD lifetime (τ_m) of MCF7 cells exposed to control media, media with 2DG at 50 mM, media without glucose, and media with cyanide.*P < 0.05, **P < 0.01, ***P < 0.001, ****P < 0.0001, ns p>0.05, for two-sided student's t-test.

2.2 Supplementary Tables

Table S1. Number of cells in each group

Cell Type	Group	Number	Image Number
MCF7 Cancer Cells	50 mM 2-DG	475	17
	Cyanide	664	17
	No Glucose	533	16
	Control	841	16
Macrophages	M0	1828	23
	M1	1074	22
	M2	1706	23

Table S2. Average prediction performance of random forest tree model trained with features of decay fitting in predicting metabolic phenotypes of macrophages in a 5-fold cross-validation.

	M0 vs M1	M0 vs M2	M1 vs M2
Accuracy	0.926	0.711	0.946
Specialty	0.917	0.689	0.950
Sensitivity	0.930	0.733	0.940
Precision	0.955	0.718	0.919
Recall	0.930	0.733	0.940

Table S3. Average prediction performance of random forest tree model trained with features of phasor analysis in predicting metabolic phenotypes of macrophages in a 5-fold cross-validation.

	M0 vs M1	M0 vs M2	M1 vs M2
Accuracy	0.897	0.641	0.924
Specialty	0.869	0.626	0.937
Sensitivity	0.913	0.659	0.907
Precision	0.924	0.620	0.905
Recall	0.913	0.659	0.907

Table S4. Average prediction performance of random forest tree model trained with features of phasor analysis and decay fitting in predicting metabolic phenotypes of macrophages in a 5-fold cross-validation.

	M0 vs M1	M0 vs M2	M1 vs M2
Accuracy	0.939	0.758	0.957
Specialty	0.932	0.756	0.964
Sensitivity	0.943	0.760	0.945
Precision	0.961	0.776	0.942
Recall	0.943	0.760	0.945

Table S5. Average prediction performance of random forest tree model trained with features of decay fitting in predicting metabolic states of cancer cells in a 5-fold cross-validation.

	Inhibit Glycolysis vs. Inhibit OXPHOS	Control vs. Inhibit Glycolysis	Control vs. Inhibit OXPHO
Accuracy	0.863	0.785	0.785
Specialty	0.830	0.777	0.774
Sensitivity	0.887	0.792	0.796
Precision	0.879	0.819	0.832
Recall	0.887	0.792	0.796

Table S6. Average prediction performance of random forest tree model trained with features of phasor analysis in predicting metabolic states of cancer cells in a 5-fold cross-validation.

	Inhibit Glycolysis vs. Inhibit OXPHOS	Control vs. Inhibit Glycolysis	Control vs. Inhibit OXPHOS
Accuracy	0.877	0.755	0.704
Specialty	0.844	0.745	0.663
Sensitivity	0.897	0.763	0.736
Precision	0.897	0.789	0.751
Recall	0.897	0.763	0.736

Table S7. Average prediction performance of random forest tree model trained with features of decay fitting and phasor analysis in predicting metabolic states of cancer cells in a 5-fold cross-validation.

	Inhibit Glycolysis vs. Inhibit OXPHOS	Control vs. Inhibit Glycolysis	Control vs. Inhibit OXPHOS
Accuracy	0.896	0.829	0.818
Specialty	0.896	0.838	0.806
Sensitivity	0.915	0.821	0.827
Precision	0.918	0.861	0.857
Recall	0.915	0.821	0.827

Table S8. P-values of two-sided t-tests for evaluation of the accuracy and ROC AUC values from the 5-fold cross-validation models for comparison of different classifiers. NS= not significant, p-value >0.05.

		Decay vs Phasor	Decay vs Decay + Phasor	Phasor vs Decay + Phasor
M0 vs M1	Accuracy	0.00057	0.00781	1.85E-05
	AUC	0.00142	0.00566	0.00073
M1 vs M2	Accuracy	NS	NS	0.01968
	AUC	0.00505	NS	1.34E-05
M0 vs M2	Accuracy	3.05E-05	0.00165	1.84E-06
	AUC	3.9E-07	0.00171	9.25E-07
Glycolysis vs OXPHOS	Accuracy	NS	NS	0.03093
	AUC	NS	0.02044	0.01212
Glycolysis vs Control	Accuracy	NS	0.00074	0.00087
	AUC	NS	0.00034	0.00011
OXPHOS vs Control	Accuracy	0.00039	0.00252	7.931E-06
	AUC	0.00032	0.01135	0.00018
